# Do Chest Compressions Benefit the Brain Beyond Restoring Circulation? ROSC-Mediated and ROSC-Independent Pathways of Bystander CPR After Out-of-Hospital Cardiac Arrest

**DOI:** 10.3390/jcm15187095

**Published:** 2026-09-13

**Authors:** Gyuchong Cho, Youdong Sohn

**Affiliations:** Department of Emergency Medicine, Kangdong Sacred Heart Hospital, Hallym University College of Medicine, Seoul 05355, Republic of Korea; emdrcho@naver.com

**Keywords:** cardiopulmonary resuscitation, out-of-hospital cardiac arrest, mediation analysis, return of spontaneous circulation, neurological outcome, neuroprotection, emergency medical services, registries

## Abstract

**Background/Objectives:** Bystander cardiopulmonary resuscitation (CPR) improves outcome after out-of-hospital cardiac arrest (OHCA), but whether its neurological benefit operates solely through prehospital return of spontaneous circulation (ROSC) is unresolved. We quantified the mediated and unmediated components. **Methods:** Retrospective cohort study of the Korean nationwide OHCA registry (2009–2024) in adults with cardiac or respiratory arrest, excluding arrests witnessed by on-duty personnel. The primary outcome was favorable neurological survival (Cerebral Performance Category 1–2 at discharge) defined across the whole cohort; prehospital ROSC was the mediator. Effects were decomposed with logistic models, including an exposure-by-mediator interaction. **Results:** Among 80,784 patients, 59,982 (74.2%) received bystander CPR. The natural direct effect was +1.24 percentage points (95% CI 0.97 to 1.51). With prehospital ROSC fixed by intervention at not achieved—for everyone, not only for patients observed to lack it—the controlled direct effect was +0.65 (0.41 to 0.85) and +0.70 (0.46 to 0.94; E-value 2.43, lower confidence limit 1.92) on restriction to 2016–2024. The total effect was +5.24 points (4.85 to 5.60), 76.4% of it ROSC-mediated. No survival effect was demonstrable at that value (+0.25, −0.14 to 0.61), and adjustment for initial rhythm left the neurological estimate indistinguishable from zero (+0.36, −0.02 to 0.69). **Conclusions:** A small component of the neurological benefit may lie outside the prehospital ROSC pathway—not outside restored circulation altogether since such survivors regained circulation in hospital—though most was ROSC-mediated. It is not established: an unmeasured confounder of moderate strength, or initial rhythm acting as a confounder rather than a mediator, would account for it. The estimates apply to events with ascertained bystander-CPR status, undocumented in 70% of otherwise eligible events. Prehospital ROSC may nonetheless understate the value of bystander response.

## 1. Introduction

Out-of-hospital cardiac arrest (OHCA) affects several hundred thousand people each year across Europe and North America, and fewer than one in ten survives to hospital discharge [1,2]. Among the determinants of that outcome, bystander cardiopulmonary resuscitation (CPR) is the one most amenable to intervention: it can be taught to the general public, prompted by emergency dispatchers, and delivered before any professional responder arrives. Its association with survival is consistent across health systems and is reflected in every major resuscitation guideline [3,4].

Less settled is the question of how that benefit reaches the patient. The chain-of-survival framework places return of spontaneous circulation (ROSC) as the pivotal intermediate step: chest compressions generate enough forward flow to make defibrillation and myocardial recovery possible, circulation returns, and survival follows [5]. On this account, restoring circulation is not merely correlated with benefit but is the route through which benefit travels.

A second possibility has long been entertained. Chest compressions generate perhaps a quarter to a third of normal cardiac output, and direct measurement during human resuscitation shows coronary perfusion pressures that are low but sufficient, above a threshold, to permit return of circulation [6]. Reports of transient consciousness during ongoing compressions indicate that cerebral perfusion can, at least occasionally, be sufficient to sustain awareness [7]. If compressions preserve neuronal viability during the no-flow interval itself, then part of the benefit of bystander CPR would be delivered before—and independently of—the restoration of circulation. The two accounts carry different practical weight. The first makes prehospital ROSC an adequate surrogate for the benefit of bystander response; the second does not and would bear on how termination-of-resuscitation rules weigh the absence of field ROSC.

Distinguishing the two requires separating the portion of the association that operates through ROSC from any portion that does not, which is the object of causal mediation analysis. Several groups have applied this framework to OHCA registries. Bessen and colleagues, studying ventricular fibrillation arrests in a metropolitan system, found that waveform measures of myocardial physiology mediated 29–53% of the survival benefit of bystander CPR [8]. Chen and colleagues, in a Beijing cohort, attributed 82.5% of the indirect effect on survival to achieving a sustained pulse on arrival [9]. Others have used the framework to decompose the effects of upstream determinants, including regional deprivation acting through delays in emergency medical services (EMS) activation in this same national registry [10], and of arrest timing in a large North American registry [11]. What none of these has established is whether any of the benefits reach neurological outcome without passing through ROSC—the outcome that matters most to patients and the one on which the two accounts diverge. A ROSC-independent component is what the neuroprotective account predicts, and the chain-of-survival account does not, so its presence or absence, rather than the size of the mediated share, is the discriminating quantity.

Outcome ascertainment bears on such an analysis. Neurological status is conventionally recorded only for patients who survive, so restricting the analysis to them conditions on a variable that bystander CPR influences; reporting it across the whole cohort, with death counted as unfavorable, is the Utstein approach and avoids this [12].

We therefore analyzed 16 years of the Korean national OHCA registry to determine whether the neurological benefit of bystander CPR includes a component that does not operate through prehospital ROSC, to estimate its size against the mediated component, and to identify which patient and system factors determine the benefit actually realized.

## 2. Materials and Methods

### 2.1. Study Design and Data Source

We conducted a retrospective cohort study using the Korean national OHCA registry maintained by the Korea Disease Control and Prevention Agency (KDCA). The registry is a government-mandated, population-based surveillance system established under Article 9 of the Cardiovascular and Cerebrovascular Disease Prevention and Management Act and is designated an approved national statistic (No. 117088). It captures every OHCA event assessed by EMS in South Korea, a country served by a single, publicly operated, fire-department-based EMS system.

Prehospital data are abstracted from standardized EMS run sheets. Hospital data are obtained by structured medical record review performed by trained abstractors at each receiving institution, following a national protocol with centralized quality assurance; variables are defined in accordance with Utstein reporting recommendations [12]. We analyzed events occurring between 1 January 2009 and 31 December 2024. Prehospital ROSC, the mediator of interest, was not among the variables collected in the registry’s inaugural year; the analytic period therefore begins in 2009. Reporting follows the STROBE statement for observational studies.

### 2.2. Participants

We included adults aged 18 years or older with EMS-assessed OHCA of presumed medical origin, restricted to cardiac and respiratory causes, who were transported to a participating hospital. We excluded events of non-medical origin (trauma, drowning, poisoning, asphyxia, burns, and electrocution), medical causes other than cardiac or respiratory, and records in which bystander CPR status, prehospital ROSC status, or any model covariate was not recorded.

Arrests witnessed or found by on-duty EMS personnel or health professionals were excluded, since bystander CPR is not defined for such events. The registry’s bystander CPR variable carries a dedicated not-applicable code for these events from 2016 onward only; in earlier years, the same events are identifiable from the witness-type fields, which distinguish on-duty from off-duty and lay witnesses throughout the study period. The exclusion was therefore applied uniformly across all years using the witness-type fields, so that the population is defined identically in every calendar year; the two definitions agreed on every record in the years in which both were available (Table S1). Off-duty EMS personnel and health professionals, and other occupational groups that the registry classifies as laypersons, were retained. Events in which witness type was unrecorded were retained; such misclassification would attenuate rather than exaggerate the estimated effect since these events carry a favorable prognosis and enter the unexposed group (Table S1).

**Figure 1:**
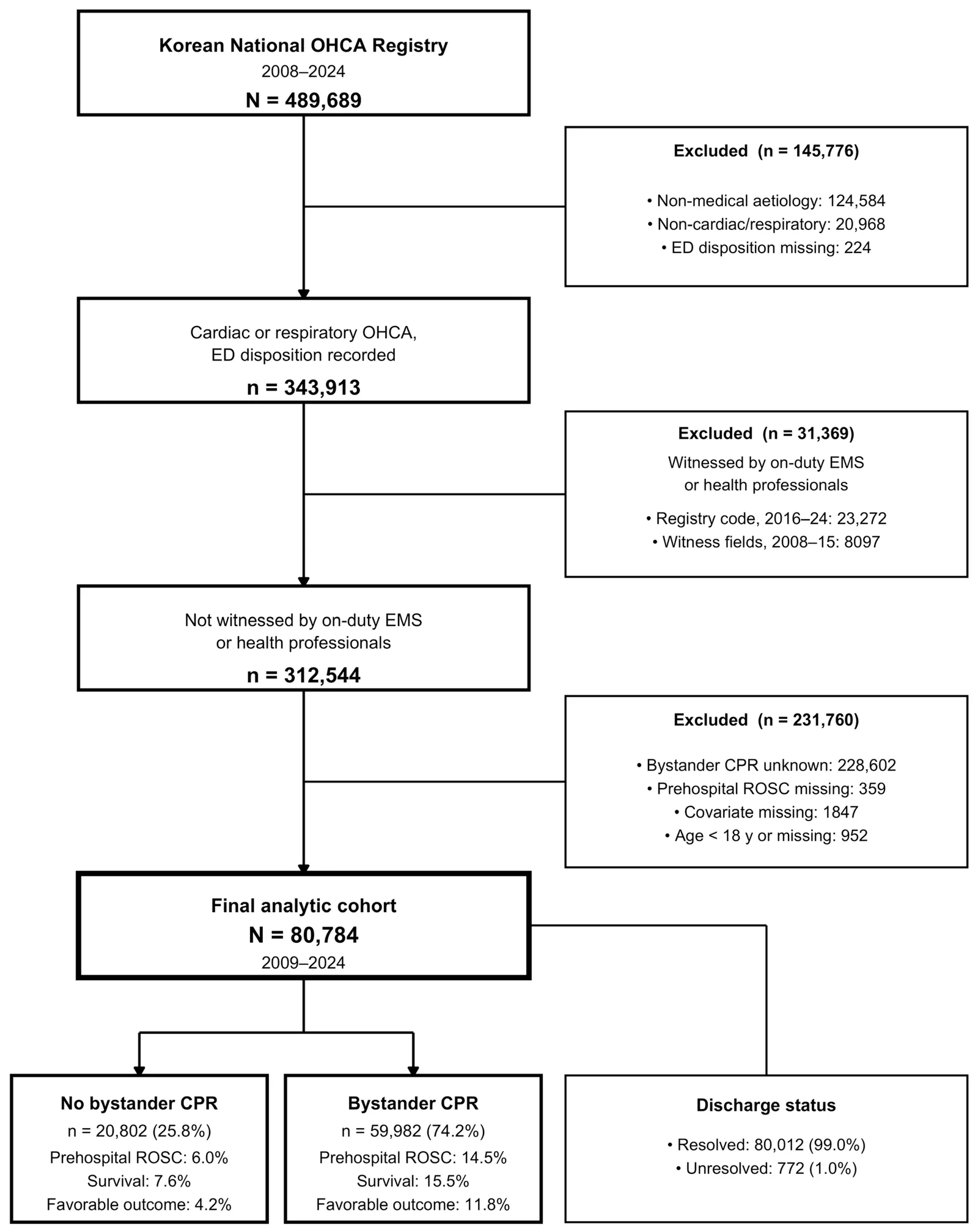
Derivation of the analytic cohort. Flow of records from the full registry to the analytic cohort. Arrests witnessed or found by on-duty emergency medical services (EMS) personnel or health professionals were excluded because bystander cardiopulmonary resuscitation (CPR) is not defined for such events; the registry carries a dedicated not-applicable code for these events from 2016 onward, and for earlier years, they were identified from the witness-type fields, which are collected throughout the study period (Table S1). Prehospital return of spontaneous circulation (ROSC) was not among the variables collected in 2008, and all 359 records excluded for missing prehospital ROSC status were from that year; the analytic period, therefore, begins in 2009. Favorable outcome denotes survival to hospital discharge with a Cerebral Performance Category of 1 or 2. Percentages in the two lower boxes are among the patients in each group whose discharge status could be resolved (59,360 of those who received bystander CPR and 20,652 of those who did not). ED, emergency department; OHCA, out-of-hospital cardiac arrest.

The derivation of the analytic cohort is shown in Figure 1.

### 2.3. Exposure, Mediator, and Outcomes

The exposure was bystander CPR, defined as chest compressions, rescue breathing, or both, initiated by a layperson before EMS arrival, and recorded as performed or not performed. The registry records this as a single variable and does not distinguish compression-only from conventional techniques.

The mediator was prehospital ROSC, defined by the registry as return of spontaneous circulation achieved before hospital arrival and sustained for at least 20 min. Circulation that returned but was not sustained for 20 min before re-arrest is recorded as ROSC not achieved. In-hospital ROSC is recorded separately since the pathway of interest concerns what bystander CPR accomplishes before professional care begins; it was reserved for a sensitivity analysis.

The primary outcome was favorable neurological survival, defined as survival to hospital discharge with a Cerebral Performance Category (CPC) of 1 or 2 [13]. The secondary outcome was survival to hospital discharge. Both outcomes were defined across the entire cohort, with death counted as an unfavorable outcome, as recommended by the Utstein templates [12]; neurological status was not analyzed only among survivors.

Because CPC is recorded only for patients who left the first hospital alive, whether by discharge or by transfer, discharge status itself was established first, using a fixed hierarchy across registry fields: emergency department disposition, in-hospital discharge result, recorded date of death, and the corresponding fields for patients transferred to a second hospital. Emergency department disposition alone would classify patients admitted alive as survivors irrespective of subsequent in-hospital death; the multi-field sequence resolves those patients correctly. Fields describing the second hospital have been collected only since 2015, so patients transferred alive in earlier years could not be resolved; unresolved status was accordingly more frequent in 2009–2014 (5.0%) than in 2015–2019 (0.65%) or 2020–2024 (0.03%). Such patients were left unresolved rather than counted as deaths. The full classification hierarchy is given in Table S2 and its resolution by year in Table S3.

### 2.4. Covariates

Models were adjusted for age (continuous), sex, arrest location (public vs. non-public), witness status, and calendar year. Calendar year was included because both bystander CPR provision and outcome improved substantially over the study period; among the measured covariates, it showed the largest imbalance between exposure groups (standardized mean difference 0.22, with witness status next at 0.17 and the remainder at or below 0.12).

### 2.5. Causal Mediation Analysis

We first describe outcomes by bystander CPR status and prehospital ROSC status without adjustment to display the joint distribution on which the subsequent decomposition rests.

The association between bystander CPR and each outcome was then decomposed into a component transmitted through prehospital ROSC and a component that was not, using a counterfactual causal mediation framework [14].

The quantities estimated are counterfactual contrasts, and it is worth stating in plain terms what each represents. The total effect is the difference in outcome that would be observed if the whole cohort received bystander CPR compared with the same cohort receiving none. The natural indirect effect is the portion of that difference attributable to the fact that bystander CPR changes the probability of achieving prehospital ROSC: it is the change in outcome that would occur if every patient received bystander CPR, but the probability of achieving ROSC was shifted from what it would be without CPR to what it would be with it. The natural direct effect is the remainder—the change in outcome that would occur if every patient received bystander CPR while the probability of achieving prehospital ROSC was held fixed at its no-CPR value. Their sum is the total effect, and the proportion mediated is the indirect effect divided by the total. The controlled direct effect is a related but distinct quantity: the effect of bystander CPR when the mediator is fixed by intervention at a specified value—here, ROSC achieved or not achieved—and it is therefore reported separately for each fixed value. Because the mediator is set by hypothetical intervention rather than conditioned upon, a controlled direct effect is not the same quantity as an effect estimated within the observed subgroup of patients who happen to have that mediator value; conditioning on an observed post-exposure mediator can itself introduce collider bias. Throughout this paper, phrases such as “with prehospital ROSC fixed at not achieved” describe this hypothetical-intervention contrast and should not be read as a claim about the subgroup of patients observed not to have achieved it.

Logistic models were fitted for the mediator given exposure and covariates and for the outcome given exposure, mediator, covariates, and an exposure-by-mediator interaction term, specified in advance so that the decomposition would not assume its absence. Natural direct and indirect effects were computed from this single pair of models by standardizing over the mediator distribution that would obtain in the absence of bystander CPR. Effects are reported on the risk-difference scale as percentage points. Controlled direct effects at each fixed value of the mediator are reported alongside since with interaction present, the averaged direct effect is a weighted combination of the contrasts at those fixed values. Path-specific odds ratios are reported for the exposure–mediator (a), mediator–outcome (b), and adjusted exposure–outcome (c′) associations.

Confidence intervals were obtained from 500 bootstrap resamples for the principal decomposition and 300 within-effect-modification strata. This number reflects the computational cost of refitting two logistic models—and, for the principal analysis, imputing unresolved outcomes—within every resample; a higher count would narrow the reported interval boundaries only marginally relative to the resolution limit described below, and 500/300 resamples were judged adequate for the point estimates and the qualitative conclusions drawn from them. Outcome status remained unresolved for 772 patients (1.0%); the principal analysis excludes them, and the consequences of every possible assignment of their outcomes are bounded separately. The smallest two-sided *p*-value obtainable from B resamples is 2/(B + 1), which is 0.004 at B = 500; estimates for which no resample crossed the null are accordingly reported as *p* < 0.004, this being a limit of resolution rather than an exact value.

### 2.6. Identifying Assumptions

The decomposition into natural direct and indirect effects carries a causal interpretation only under four conditions, stated explicitly here because they, rather than the choice of model, determine what the estimates mean:

- (A1) No unmeasured confounding of the exposure–outcome relation, conditional on the measured covariates.
- (A2) No unmeasured confounding of the mediator–outcome relation, conditional on exposure and covariates.
- (A3) No unmeasured confounding of the exposure–mediator relation, conditional on covariates.
- (A4) No confounder of the mediator–outcome relation that is itself affected by the exposure (the cross-world independence condition).

Assumptions A1 and A3 concern who receives bystander CPR; both are addressed by adjustment and by the weighting analysis described below, and their residual violation is quantified by the E-values reported in Section 3.6. Assumption A2 concerns who achieves prehospital ROSC among those with the same exposure and covariates. Assumption A4 is the most restrictive and is not satisfied by initial cardiac rhythm, which is recorded after compressions have begun and may be influenced by them; analyses adjusted for rhythm are therefore reported as a conservative lower bound rather than as corrected estimates and are discussed as such in Section 4.

### 2.7. Effect Modification

Effect modification was assessed for three factors, chosen because they differ in where they sit relative to the exposure. Time from collapse to EMS activation precedes bystander CPR; it was derived from fire service timestamps available since 2013, restricted to witnessed arrests, and computed after the exclusions enumerated in Section 3.7. Pre-arrival dispatcher instruction is a determinant of bystander CPR. Initial cardiac rhythm recorded by EMS is measured after bystander CPR has begun and may be influenced by it. For each factor, we tested a two-way interaction with bystander CPR and a three-way interaction with bystander CPR and ROSC by likelihood-ratio test and repeated the decomposition within strata. Because absolute and relative effect measures can diverge when baseline risk differs across strata, both are reported, and numbers needed to treat are derived from risk differences.

### 2.8. Sensitivity and Robustness Analyses

Robustness was examined in four ways.

First, by restricting the analysis to 2016 onward, the period in which the registry itself assigns a not-applicable code to arrests witnessed by on-duty EMS personnel or health professionals, so that the population definition rests on the registry coding alone rather than on the reconstruction described in Section 2.2. This is the principal robustness analysis for the direct effect.

Second, by E-values on the risk-ratio scale for the controlled direct effect with prehospital ROSC fixed at not achieved, computed at the point estimate and at the lower confidence limit [15]. An E-value states the minimum strength of association, on the risk-ratio scale, that an unmeasured confounder would need to have with both the exposure and the outcome in order to explain away the observed estimate.

Third, by additional adjustment for initial rhythm in the subcohort in which it was recorded—a variable measured after exposure onset, which therefore violates assumption A4 and yields a conservative lower bound rather than a corrected value.

Fourth, by inverse probability of treatment weighting, in which a propensity model for bystander CPR was fitted on the same covariates, stabilized weights were truncated at the 1st and 99th percentiles, and the total effect was re-estimated with and without additional regression adjustment (Table S4). The weighting approach relies on correct specification of the exposure model rather than of the outcome model and therefore tests whether the total effect depends on a particular regression form; it bears only on confounding of the exposure–outcome relation and not on the identification of the mediated and direct components.

Analyses were performed in R version 4.2.3, with weighted models fitted using the survey package (version 4.2-1). A large language model (Claude; Anthropic, San Francisco, CA, USA; https://claude.ai) was used to assist with statistical programming and with language editing; it was not used to generate data, to design the study, or to interpret results. All analytic decisions and all interpretations are the authors’. The decomposition was implemented directly from the fitted logistic models described above rather than with a packaged routine, so that the interaction-consistent standardization and the imputation of unresolved outcomes could be carried inside the same bootstrap.

## 3. Results

### 3.1. Cohort

Of 489,689 EMS-assessed OHCA records, 365,105 were of presumed medical origin, and 344,137 had a cardiac or respiratory cause; emergency department disposition was recorded for 343,913. Excluding 31,369 arrests witnessed or found by on-duty EMS personnel or health professionals left 312,544 events. Bystander CPR status was recorded for 83,942 of these, prehospital ROSC status for 83,583, and all model covariates for 81,736; restriction to adults yielded the analytic cohort of 80,784 patients from 2009 to 2024 (Figure 1).

The largest single exclusion was the 228,602 events for which bystander CPR status was recorded as unknown; of these, 190,853 also had prehospital ROSC status, all covariates, and adult age recorded and are therefore the appropriate comparison group for the analytic cohort in the bias analysis of the Limitations subsection and Table S5, since they are eligible on every criterion other than exposure ascertainment. Discharge status was resolved for 80,012 patients (99.0%); the remaining 772 (1.0%) could not be classified and were excluded from the decomposition, with the consequences of that exclusion bounded in the sensitivity analyses (Section 3.6).

### 3.2. Patient Characteristics

Bystander CPR was performed in 59,982 patients (74.2%) and not performed in 20,802 (25.8%) (Table 1). Patients receiving bystander CPR were younger (mean 68.4 [SD 15.9] vs. 70.3 [15.0] years), more often arrested in a public location (21.1% vs. 17.0%), and more often had the arrest witnessed (60.2% vs. 51.9%); sex distribution was almost identical (66.0% vs. 66.4% male). Prehospital ROSC was achieved in 8859 patients receiving bystander CPR (14.8%) and 1243 who did not (6.0%).

### 3.3. Joint Distribution of Bystander CPR, Prehospital ROSC, and Outcome

Table 2 shows outcomes by bystander CPR and prehospital ROSC status without adjustment. Three features are apparent.

First, bystander CPR was associated with a substantially higher probability of achieving prehospital ROSC: 14.52% vs. 5.98%. Second, achieving prehospital ROSC dominated outcome: favorable neurological survival occurred in 68.40% of patients who achieved it and 1.64% of those who did not. Third—and bearing directly on the question posed—within the stratum that never achieved prehospital ROSC (70,157 patients, or 87.7% of those with resolved status), favorable neurological survival was nonetheless more frequent with bystander CPR than without (1.8% vs. 1.1%); within the stratum achieving ROSC, the corresponding figures were 70.5% and 53.4%.

### 3.4. The Component Not Transmitted Through Prehospital ROSC

For favorable neurological survival, a component of the effect of bystander CPR did not operate through prehospital ROSC: the natural direct effect was +1.24 percentage points (95% CI 0.97 to 1.51) (Table 3). The remainder, +4.01 points (3.70 to 4.28), was transmitted through the mediator, so that the total effect was +5.24 points (4.85 to 5.60) and the proportion mediated 76.4% (72.2 to 80.6). For survival to discharge, the direct component was smaller, +0.69 points (0.34 to 1.04) against +4.58 points (4.24 to 4.91) transmitted through prehospital ROSC, giving a total effect of +5.27 points (4.83 to 5.75) and a proportion mediated of 86.8% (81.3 to 93.1).

On the odds-ratio scale, the exposure–mediator association was 2.22 for both outcomes. The mediator–outcome association, estimated in patients not receiving bystander CPR, was 79.98 for neurological outcome and 51.69 for survival. The exposure-by-mediator interaction was modest and not distinguishable from the null for neurological outcome (1.19, 0.98, to 1.44) and larger for survival (1.49, 1.24, to 1.75).

### 3.5. Direct Effects with the Mediator Fixed by Intervention

Because the averaged direct effect is a weighted combination of contrasts at each fixed value of the mediator, controlled direct effects are reported for each value separately. For favorable neurological survival, the controlled direct effect was +0.65 percentage points (0.41 to 0.85) with prehospital ROSC fixed at not achieved and +10.31 points (7.89 to 12.74) with it fixed at achieved; weighted by the mediator distribution that would obtain without bystander CPR, in which about 93% of patients do not achieve prehospital ROSC, the two fixed values contribute in approximately equal measure to the averaged direct effect.

For survival to discharge, the corresponding values were +0.25 points (−0.14 to 0.61; *p* = 0.20) and +8.86 points (5.97 to 11.71). The controlled direct effect on survival with ROSC fixed at not achieved was therefore not statistically distinguishable from zero. Under that same fixed value, survival with an unfavorable neurological outcome was less frequent with bystander CPR (−0.28 points, −0.53 to −0.02), so the gain in favorable neurological survival reflects a shift in the outcome among survivors rather than an increase in their number.

### 3.6. Robustness of the Estimates

Restricting the analysis to 2016 onward, the period in which the registry itself assigns a not-applicable code to arrests witnessed by on-duty personnel so that the population definition does not depend on the reconstruction described in Section 2.2 gave 65,318 patients (Table 4) and reproduced the pattern with larger direct components: the direct effect was +1.40 points (1.12 to 1.69) with a proportion mediated of 73.1%, and the controlled direct effect without prehospital ROSC was +0.70 points (0.46 to 0.94). The E-value for that estimate was 2.43, with a lower confidence limit of 1.92; for the full cohort, the corresponding values were 2.23 and 1.81.

Initial cardiac rhythm has been recorded since 2015 and was classifiable in 66,937 patients (Table 4). It is measured after bystander CPR has begun and is therefore an exposure-induced confounder of the mediator–outcome relation, violating assumption A4, so estimates adjusted for it are reported as a conservative lower bound. Adding rhythm to the outcome model significantly improved its fit for both outcomes (likelihood-ratio test against the model without it: χ^2^(4) = 2391.9, *p* < 0.001 for favorable neurological survival; χ^2^(4) = 2035.7, *p* < 0.001 for survival to discharge), confirming that the shift in the estimates below reflects a variable carrying substantial information rather than a negligible one. Under that adjustment, the controlled direct effect on neurological outcome with ROSC fixed at not achieved retained its magnitude but was no longer distinguishable from zero (+0.36 points, −0.02 to 0.69; *p* = 0.064), while the corresponding effect on survival moved below zero (−0.60 points, −1.10 to −0.14; *p* = 0.016). The natural direct effect on neurological outcome was +0.67 points (0.28 to 0.99).

In-hospital ROSC was recorded for 33,292 patients in the stratum without prehospital ROSC (Table 4). Bystander CPR did not predict it (42.2% vs. 43.1%), and fixing it left the controlled direct effect unchanged at +0.62 points, with positive estimates whether or not it was achieved.

Assignment of the 772 patients with unresolved discharge status did not materially affect the estimates (Table 4, Panel F). With the same proportion assigned a favorable outcome in both exposure groups, the direct effect on neurological outcome ranged from +1.12 to +1.37 points. Only assignment differing in direction between the groups reduced it to +0.33 points and to +1.30 points on restriction to 2016–2024; that assignment runs opposite to the difference observed among resolved emergency department survivors.

Before weighting, calendar year showed the largest imbalance between exposure groups (standardized mean difference 0.22), followed by witness status (0.17), age (0.12), and arrest location (0.10); after inverse probability of treatment weighting, all standardized differences were below 0.02. Total-effect odds ratios from weighting were consistent with those from regression adjustment (Table 4). Both models use the same functional form for continuous covariates, so this comparison bears on the outcome model rather than on that form.

### 3.7. Factors Modifying the Magnitude of Benefit

**Figure 2:**
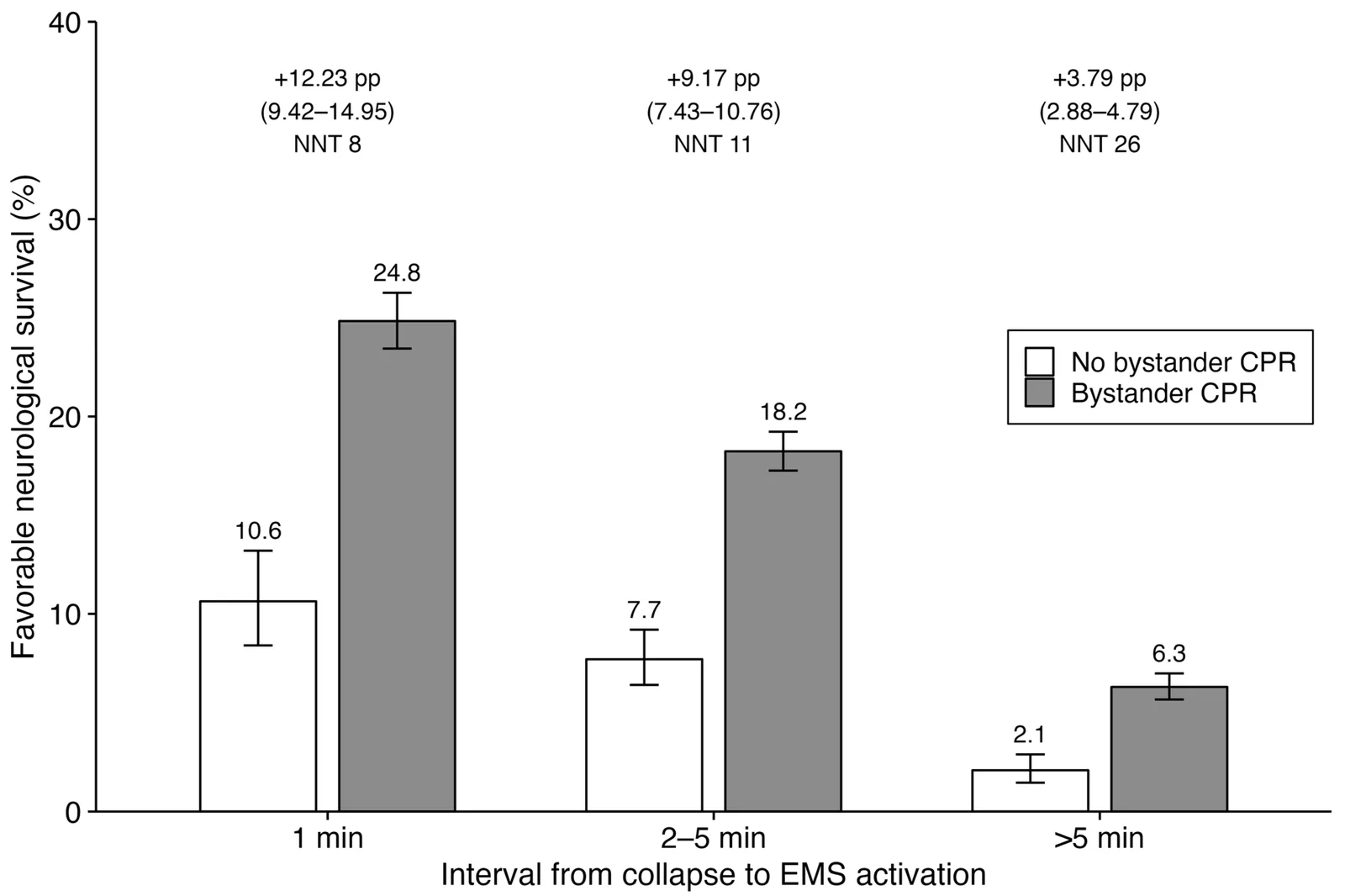
Favorable neurological survival by interval from collapse to EMS activation. Unadjusted proportion of patients achieving favorable neurological survival by bystander cardiopulmonary resuscitation (CPR) status and interval from collapse to emergency medical services (EMS) activation, among witnessed arrests from 2013 to 2024 with a recorded interval greater than zero (*n* = 19,081). Error bars are exact binomial 95% confidence intervals. Figures above each pair give the covariate-adjusted total effect on the risk-difference scale with its bootstrap confidence interval and the corresponding number needed to treat. Absolute benefit declines steeply as the interval lengthens, while the effect on the logit scale does not vary across intervals (likelihood-ratio test *p* = 0.87); the gradient therefore reflects a falling baseline probability of a favorable outcome rather than compressions becoming less effective when notification is delayed. Definitions of the interval and the exclusions applied are given in the Table 5 footnote.

The interval from collapse to EMS activation could be calculated for witnessed arrests from 2013 onward; after the exclusions listed in Table 5, 19,081 patients remained. Absolute benefit fell markedly as the interval lengthened (Table 5, Figure 2). For favorable neurological survival, the total effect was +12.23 percentage points (9.42 to 14.95) at 1 min, corresponding to 8 patients needing to receive bystander CPR for one additional favorable outcome; +9.17 points (7.43 to 10.76) at 2 to 5 min (11 patients); and +3.79 points (2.88 to 4.79) beyond 5 min (26 patients). Values for survival to discharge were 10, 11, and 33 patients. Retaining the 14,876 witnessed arrests with a zero-minute interval did not alter the gradient; as detailed in the Table 5 footnote, neither bystander-CPR status nor outcome was concentrated in a single cell among them.

This gradient was confined to the absolute scale. There was no evidence of interaction between bystander CPR and the interval on the logit scale for either outcome (likelihood-ratio test *p* = 0.87 and *p* = 0.30), indicating that the relative effect was stable while the baseline probability of a favorable outcome declined.

Pre-arrival dispatcher instruction, examined as a negative control, showed no evidence of modifying the association with either outcome (likelihood-ratio test *p* = 0.46 for survival and *p* = 0.64 for neurological outcome; *n* = 72,661).

## 4. Discussion

In a national cohort of 80,784 adults with OHCA of cardiac or respiratory origin, part of the neurological benefit of bystander CPR appeared to lie outside the pathway through prehospital ROSC. The natural direct effect was 1.24 percentage points, a controlled direct effect was present when prehospital ROSC was fixed by intervention at not achieved, and it was larger in the subcohort from 2016 onward in which the registry itself identifies arrests witnessed by on-duty personnel. Most of the benefit—4.01 of 5.24 points, or 76.4%—was nonetheless transmitted through prehospital ROSC, as was the case for survival to discharge (86.8%). Both outcomes, therefore, showed partial mediation. These estimates should be read as conditional on an ascertained bystander-CPR status: that status was undocumented for 70% of otherwise eligible events, and documentation itself varied by calendar year, region, and dispatch center (see Limitations).

The larger share travels through ROSC is close to arithmetically guaranteed and is evident without any modeling. Bystander CPR was associated with a 2.4-fold higher probability of achieving prehospital ROSC (14.52% vs. 5.98%), and achieving it separated outcomes almost categorically—favorable neurological survival occurred in 68.40% of patients who did and 1.64% of those who did not. Given a mediator that is this strongly associated with outcome, an intervention that raises the probability of reaching it will transmit most of its benefit that way. The discriminating quantity is therefore not the mediated fraction, which is close to a structural consequence of the pathway, but whatever remains outside it.

That residual component was present at both fixed values of the mediator. Raw outcomes already point the same way: among the 70,157 patients (87.7% of those with resolved status) who did not achieve prehospital ROSC, favorable neurological survival was more frequent with bystander CPR than without (Table 2). The controlled direct effect—the contrast that would be expected if prehospital ROSC were fixed by intervention at not achieved for the whole cohort, rather than the effect observed within this subgroup—was 0.65 percentage points (95% CI 0.41 to 0.85) and 10.31 points with ROSC fixed at achieved. Because the value “not achieved” describes roughly 93% of the mediator distribution that would obtain in the absence of bystander CPR, it contributes approximately half of the averaged direct effect despite the smaller per-patient contrast.

This distribution bears on the competing accounts set out at the outset. If benefit were delivered solely by raising the probability of ROSC, no controlled direct effect would be expected when ROSC is fixed at not achieved. A positive controlled direct effect was present under precisely that fixed value, was larger in the 2016–2024 subcohort (0.70 points, 95% CI 0.46 to 0.94); this is compatible with compressions conferring benefit that does not depend on early restoration of circulation. The interpretation requires one qualification that the design makes explicit: prehospital ROSC was the mediator, and patients without it who survived with a favorable outcome necessarily regained circulation in the hospital. The direct pathway is therefore compatible with a component independent of ROSC, specifically during the prehospital interval, not with neurological survival independent of restored circulation altogether. A plausible reading is that compressions preserved cerebral viability during the low-flow period, so that patients in whom circulation was later restored arrived at that point in better neurological condition—consistent with reports of preserved consciousness during ongoing compressions, which requires cerebral perfusion adequate to sustain awareness [7].

Initial rhythm admits two readings that lead to opposite conclusions, and these data cannot decide between them. On the first, rhythm is a confounder of the ROSC–outcome relation that happens to be measured after exposure onset: patients who received bystander CPR are more often found in a shockable rhythm for reasons that independently predict outcome, in which case the rhythm-adjusted estimate is the more credible one and the unadjusted controlled direct effect is inflated. Under that reading, the direct component on neurological outcome is not distinguishable from zero (+0.36 points, −0.02 to 0.69), the corresponding effect on survival is negative, and what is described here as a residual pathway would instead be residual confounding. On the second, rhythm is itself a step from compressions to outcome—compressions that maintain or restore a shockable rhythm act through it—so that adjusting for rhythm subtracts genuine mediated effect and the loss of significance under that adjustment (Section 3.6) reflects over-adjustment rather than the absence of a direct pathway. Because the registry records rhythm only after compressions have begun, nothing in these data adjudicates between a confounder and a further mediator, and neither reading can be given precedence over the other. The rhythm-adjusted estimate is therefore reported as a lower bound, and the confounded reading is treated as no less tenable than the mediated one; the direct component is a finding compatible with these data rather than one established by them.

The two outcomes diverged in a way that supports rather than undermines this reading. With ROSC fixed at not achieved, the controlled direct effect on survival to discharge was 0.25 points (95% CI −0.14 to 0.61); it cannot be distinguished from no effect. Neurological benefit without a corresponding survival benefit at that same fixed value would be expected if compressions altered the quality of recovery among those who ultimately survived rather than the probability of survival itself, although the two estimates were not formally compared.

These estimates sit alongside the small existing literature. Bessen and colleagues found that waveform measures of myocardial physiology mediated 29–53% of the survival benefit of bystander CPR in ventricular fibrillation arrests [8], and Chen and colleagues, using path analysis in a Beijing cohort, attributed 82.5% of the indirect association with survival to the survived-event pathway [9]. That figure is a share of the indirect association rather than of the total, so it is not directly comparable with the proportions reported here. Work in this same registry used the interval from collapse to EMS activation as a mediator of regional deprivation [10] rather than, as here, as a modifier. The proportion mediated reported here, 76.4%, follows largely from the strength of the exposure–mediator and mediator–outcome associations rather than from anything specific to bystander CPR; what bears on the competing accounts is that its complement is not zero.

The clinical implication lies in the absolute rather than the relative scale. The number needed to treat for the total effect, overall, was 19 for favorable neurological survival and 19 for survival to discharge; among witnessed arrests, the neurological number needed to treat fell from 8 when EMS was activated within 1 min of collapse to 26 beyond 5 min, and the corresponding survival number needed to treat fell from 10 to 33. There was no evidence of interaction on the logit scale (*p* = 0.87), so the relative effect was stable, and the gradient reflects a collapsing baseline probability of a favorable outcome rather than compressions becoming less effective. The message is not that late-recognized arrests should be treated differently, but that the yield of any episode depends on how quickly the emergency was recognized and reported. A residual component outside the ROSC pathway also implies that prehospital ROSC understates the benefit of bystander response, which bears on its use as a trial endpoint. Any implication for rules that terminate resuscitation when field ROSC is absent is hypothesis-generating only: this analysis identifies a mechanistic pathway and does not evaluate the safety or effectiveness of changing termination-of-resuscitation criteria.

### Limitations

First, and most consequential for how these results should be read, bystander CPR status was unknown for 190,853 otherwise eligible events (70% of the 271,637 that met every other cohort criterion). The registry records “not performed” only where the absence is explicitly documented and “unknown” where no entry exists, so unknown status is not missing at random but weighted toward non-performance. Documentation was strongly associated with prognosis: excluded events achieved prehospital ROSC in 4.5% of cases against 12.5% in the cohort (Table S5). Documentation also varied by locality: undocumented status ranged from 49.5% in Seoul to 87.7% in Jeonnam Province across the 17 first-level administrative regions, was somewhat more common outside the eight metropolitan cities (74.5%) than within them (63.9%), and varied far more sharply across individual fire-service dispatch jurisdictions (interquartile range 64.1% to 86.1%, full range 13.2% to 96.8%, across 262 jurisdictions with at least 100 eligible events; Table S5, Panel B). This pattern is consistent with local and administrative differences in abstraction practice, but it could equally reflect systematic differences in regional or dispatch-level resource levels—for instance, staffing, training, or workload in less-resourced jurisdictions—that plausibly relate to prognosis as well as to documentation; the data cannot distinguish between these explanations. It is presented descriptively since a formal model of documentation was not fitted, but it indicates that non-random missingness operates at a finer grain than calendar year alone and is unlikely to be fully captured by the covariates available here—age, sex, arrest location, witness status, and calendar year—none of which encodes the jurisdiction in which an event was documented. Estimates should therefore be read as conditional on an ascertained bystander-CPR status rather than as applying uniformly across jurisdictions. Because exposure is the only variable missing in these events, their exclusion can be bounded rather than assumed: across every assignment of their exposure, the direct effect remained positive, from +1.18 points if none received bystander CPR to +0.21 if all did, whereas the total effect fell to between +1.14 and +4.05 points. Bounding of this kind constrains what the excluded events could imply about the direct effect; it does not make the analysis representative of them. The estimates apply to events with an ascertained bystander response, and neither the total nor the direct effect should be read as a value for the full eligible population.

Second, the no-flow time before compressions began is not recorded. Time to EMS activation is an imperfect proxy: the collapse time is estimated rather than observed, and that analysis is confined to witnessed arrests from 2013 onward. The registry also does not distinguish compression-only from conventional technique, and EMS response interval is not captured, so neither could be examined.

Third, initial rhythm is measured after exposure onset and is plausibly influenced by it. It is therefore an exposure-induced confounder of the mediator–outcome relation, violating assumption A4, under which natural direct and indirect effects are not identified by the mediation formula; the decomposition should be read as holding under the stated assumptions rather than as established. If rhythm also lies on the pathway from compressions to outcome, adjusting for it removes genuine mediated effect along with confounding, and the adjusted estimates are then a lower bound rather than corrected values. At that bound, the controlled direct effect on neurological outcome with ROSC fixed at not achieved retained its magnitude but was no longer distinguishable from zero (+0.36 points, −0.02 to 0.69), and the corresponding effect on survival moved below zero. The direct component is therefore robust to the population definition but not to adjustment for a post-exposure variable. Whether that adjustment removes confounding or removes genuine mediated effect cannot be determined from these data; Section 4 sets out both readings and gives neither precedence.

Fourth, identification requires assumptions A1 to A4 in Section 2.6. For the controlled direct effect with prehospital ROSC fixed at not achieved, the E-value was 2.23 in the full cohort and 2.43 in the 2016–2024 subcohort, with lower confidence limits of 1.81 and 1.92; an unmeasured factor would therefore have to be associated with both bystander CPR and outcome by a risk ratio of roughly two to account for these estimates. Associations of that magnitude cannot be excluded for factors that were not used here, including the promptness of arrest recognition and the interval before compressions began. Comorbidity is recorded in the registry but was left unknown in 44% to 74% of the cohort, depending on the condition, and was therefore not used. Agreement between regression adjustment and inverse probability weighting addresses the functional form of the outcome model, but not unmeasured confounding, and the direct effects should be regarded as compatible with, rather than demonstrating, a non-ROSC pathway.

Sex was included as a covariate, but no sex-stratified decomposition was undertaken since no sex-specific hypothesis was posed for the mechanistic question addressed here and the exposure groups were almost identical in sex distribution (66.0% vs. 66.4% male); sex differences in bystander response are an established question in their own right and are better addressed by a design directed at them.

Fifth, the cohort comprises patients assessed and transported by a single national, publicly operated EMS system; patients who died at the scene without transport, including those in whom resuscitation was terminated in the field, are not represented, and the findings cannot be extended to them. Discharge status remained unresolved for 772 patients (1.0%), all of whom survived the emergency department, and most of whom had received bystander CPR (622 of 772). Estimates were insensitive to how many were assumed to have survived, and the direct effect on neurological outcome remained positive under every assignment examined, including one differing in direction between the exposure groups (Table 4, Panel F).

## 5. Conclusions

A small positive controlled direct effect on neurological outcome was present when prehospital ROSC was fixed by intervention at not achieved. It is compatible with—but does not establish—a component of benefit independent of prehospital ROSC, specifically rather than of restored circulation altogether since patients without prehospital ROSC who survived favorably necessarily regained circulation in hospital. This component was larger in the period in which the registry itself identifies arrests witnessed by on-duty personnel. It was not, however, accompanied by a demonstrable effect on survival to discharge at the same fixed value, and it did not persist once initial rhythm was included in the model. Whether that adjustment removes confounding or removes genuine mediated effect cannot be settled with these data, and the confounded reading—under which the direct component would not be distinguishable from zero—is no less tenable than the mediated one. An unmeasured factor associated with both bystander CPR and outcome by a risk ratio of about two would likewise account for the estimate (E-value 2.23 in the full cohort and 2.43 in 2016–2024, with lower confidence limits of 1.81 and 1.92). Most of the association—76.4%—was transmitted through prehospital ROSC. The estimates were obtained among the 30% of otherwise eligible events whose bystander-CPR status was documented; they apply to events with an ascertained bystander response and not to the full eligible population. Chest compressions may therefore benefit the brain beyond their effect on prehospital ROSC, but the evidence assembled here is consistent with that possibility rather than demonstrating it, and it has to be weighed against the more conservative explanations set out above. On the same reading, prehospital ROSC may understate the benefit of bystander response, which bears on its use as a trial endpoint; any implication for termination-of-resuscitation rules is hypothesis-generating only since this analysis does not evaluate the safety or effectiveness of changing those criteria. The absolute benefit of bystander CPR varied more than 3-fold with the interval from collapse to EMS activation while the relative effect did not, locating the largest practical gains in earlier recognition and reporting of cardiac arrest.

## Figures and Tables

**Table 1:** Characteristics of the analytic cohort by bystander CPR status.

Characteristic	Bystander CPR (*n* = 59,982)	No Bystander CPR (*n* = 20,802)
Age, y, mean (SD)	68.4 (15.9)	70.3 (15.0)
Age group, *n* (%)		
18–39 y	3025 (5.0)	745 (3.6)
40–59 y	13,902 (23.2)	4152 (20.0)
60–79 y	25,393 (42.3)	9339 (44.9)
≥80 y	17,662 (29.4)	6566 (31.6)
Male sex, *n* (%)	39,567 (66.0)	13,807 (66.4)
Public location, *n* (%)	12,652 (21.1)	3532 (17.0)
Arrest witnessed, *n* (%)	36,086 (60.2)	10,791 (51.9)
Prehospital ROSC, *n* (%)	8859 (14.8)	1243 (6.0)

CPR, cardiopulmonary resuscitation; ROSC, return of spontaneous circulation; SD, standard deviation. Percentages are column percentages. Prehospital ROSC is shown as a cohort characteristic here and is treated as the mediator in all subsequent analyses. Significance tests are not reported for baseline comparisons since, with a cohort this size, even trivial imbalances are statistically significant; covariate balance between exposure groups is instead quantified by standardized mean differences (Section 2.4), which are reported again before and after weighting in Table S4.

**Table 2:** Outcomes by bystander CPR and prehospital ROSC status, without adjustment.

Bystander CPR	Prehospital ROSC	*N*	Survival to Discharge, *n* (%)	Favorable Neurological Survival, *n* (%)
Yes	Achieved	8621	7063 (81.9)	6082 (70.5)
No	Achieved	1234	864 (70.0)	659 (53.4)
Yes	Not achieved	50,739	2149 (4.2)	937 (1.8)
No	Not achieved	19,418	713 (3.7)	216 (1.1)

CPR, cardiopulmonary resuscitation; ROSC, return of spontaneous circulation. Restricted to the 80,012 patients (99.0%) with resolved discharge status. Prehospital ROSC was achieved by 14.52% of patients receiving bystander CPR and 5.98% of those who did not. Favorable neurological survival occurred in 68.40% of patients achieving prehospital ROSC and 1.64% of those who did not. Favorable neurological survival denotes survival to hospital discharge with a Cerebral Performance Category of 1 or 2, with death counted as an unfavorable outcome across the whole cohort.

**Table 3:** Decomposition of the association between bystander CPR and outcome.

Component	Favorable Neurological Survival	Survival to Discharge
**Risk-difference scale, percentage points (95% CI)**		
Total effect	+5.24 (4.85 to 5.60)	+5.27 (4.83 to 5.75)
Natural direct effect, not through prehospital ROSC	+1.24 (0.97 to 1.51)	+0.69 (0.34 to 1.04)
Natural indirect effect, through prehospital ROSC	+4.01 (3.70 to 4.28)	+4.58 (4.24 to 4.91)
Proportion mediated, %	76.4 (72.2 to 80.6)	86.8 (81.3 to 93.1)
**Controlled direct effect, mediator fixed by intervention, percentage points (95% CI)**		
Prehospital ROSC not achieved	+0.65 (0.41 to 0.85)	+0.25 (−0.14 to 0.61) †
Prehospital ROSC achieved	+10.31 (7.89 to 12.74)	+8.86 (5.97 to 11.71)
**Path-specific odds ratios (95% CI)**		
Exposure–mediator (a)	2.22	2.22
Mediator–outcome (b) ‡	79.98	51.69
Exposure–outcome, adjusted for mediator (c′) §	1.47	1.06
Exposure × mediator interaction	1.19 (0.98 to 1.44)	1.49 (1.24 to 1.75)

CI, confidence interval; CPR, cardiopulmonary resuscitation; ROSC, return of spontaneous circulation. Restricted to the 80,012 patients (99.0%) with resolved discharge status. All models were adjusted for age, sex, arrest location, witness status, and calendar year. Natural direct and indirect effects were computed from a common pair of models—one for the mediator and one for the outcome, the latter including an exposure-by-mediator interaction—by standardizing over the mediator distribution that would obtain in the absence of bystander CPR; the total effect is their sum. Confidence intervals are from 500 bootstrap resamples, from which the smallest obtainable two-sided *p*-value is 2/(B + 1) = 0.004. No resample crossed the null for the total, indirect, or direct effects or for the controlled direct effect with ROSC fixed at achieved, for either outcome; these are therefore reported as *p* < 0.004, a limit of resolution rather than an exact value. † *p* = 0.20; the only component not distinguishable from zero. ‡ Estimated from the interaction model and therefore representing the association between prehospital ROSC and outcome among patients who did not receive bystander CPR. § Likewise estimated from the interaction model and, therefore, representing the exposure–outcome association among patients who did not achieve prehospital ROSC. Bold entries are section headings within the table.

**Table 4:** Sensitivity analyses of the mediation estimates.

Analysis	Favorable Neurological Survival	Survival to Discharge
**A. Restriction to 2016–2024** (*n* = 65,318)		
Total effect, pp (95% CI)	+5.20 (4.76 to 5.67)	—
Natural indirect effect, pp (95% CI)	+3.80 (3.43 to 4.15)	—
Natural direct effect, pp (95% CI)	+1.40 (1.12 to 1.69)	—
Proportion mediated, %	73.1 (68.5 to 77.5)	—
Controlled direct effect, ROSC not achieved, pp (95% CI)	+0.70 (0.46 to 0.94)	—
Controlled direct effect, ROSC achieved, pp (95% CI)	+10.58 (7.95 to 13.27)	—
E-value for controlled direct effect, ROSC not achieved	2.43 (lower confidence limit 1.92)	—
**B. E-values, full cohort**		
Controlled direct effect, ROSC not achieved	2.23 (lower limit 1.81)	1.30 (lower limit 1.06)
Controlled direct effect, ROSC achieved	1.85 (lower limit 1.68)	1.58 (lower limit 1.43)
**C. Adjustment for initial rhythm** (*n* = 66,937; 2015–2024)		
Natural direct effect, pp (95% CI)	+0.67 (0.28 to 0.99)	−0.19 (−0.61 to 0.23)
Proportion mediated, %	73.2	109.7
Controlled direct effect, ROSC not achieved, pp (95% CI)	+0.36 (−0.02 to 0.69), *p* = 0.064	−0.60 (−1.10 to −0.14), *p* = 0.016
Controlled direct effect, ROSC achieved, pp (95% CI)	+3.74 (1.46 to 5.85)	+3.68 (0.88 to 7.15)
E-value for rhythm-adjusted effect, ROSC not achieved	1.61 (lower limit 1.11)	1.48 (lower limit 1.20)
**D. In-hospital ROSC, stratum without prehospital ROSC** (*n* = 33,292)		
In-hospital ROSC achieved, %	42.2 with bystander CPR vs. 43.1 without	—
Controlled direct effect, without fixing in-hospital ROSC, pp (95% CI)	+0.62 (0.34 to 0.90)	—
Controlled direct effect, with in-hospital ROSC fixed, pp (95% CI)	+0.62 (0.38 to 0.90)	—
In-hospital ROSC achieved	+0.71 (0.27 to 1.11)	—
In-hospital ROSC not achieved	+0.55 (0.13 to 0.90)	—
Survival with an unfavorable neurological outcome, pp (95% CI) †	−0.28 (−0.53 to −0.02)	—
**E. Inverse probability of treatment weighting** (*n* = 80,012)		
Total effect, odds ratio—regression adjustment	2.51 (2.32 to 2.71)	1.85 (1.74 to 1.96)
Total effect, odds ratio—weighted	2.29 (2.14 to 2.44)	1.77 (1.68 to 1.86)
Total effect, odds ratio—weighted with additional regression adjustment	2.47 (2.31 to 2.64)	1.85 (1.74 to 1.95)
**F. Bounding of unresolved discharge status** (*n* = 80,784)		
Total effect, complete case, 772 patients excluded, pp	+5.24	+5.27
Total effect, non-differential assignment, none to all favorable, pp	+5.16 to +5.54	+5.11 to +5.61
Total effect, differential assignment against bystander CPR, pp	+4.39	+4.39
Restricted to 2016–2024	+5.12	+5.01
Natural direct effect, complete case, pp	+1.24	+0.69
Natural direct effect, non-differential assignment, pp	+1.12 to +1.37	+0.51 to +0.88
Natural direct effect, differential assignment against bystander CPR, pp	+0.33	−0.23
Restricted to 2016–2024	+1.30	+0.61
Controlled direct effect, ROSC not achieved, complete case, pp	+0.65	+0.25
Controlled direct effect, differential assignment against bystander CPR, pp	−0.28	−0.63
Restricted to 2016–2024	+0.62	+0.17

CI, confidence interval; pp, percentage points; ROSC, return of spontaneous circulation. **Panel A.** The period from which the registry assigns a dedicated not-applicable code to arrests witnessed by on-duty emergency medical services personnel or health professionals, so that the population definition rests on the registry coding alone and does not depend on the reconstruction described in Section 2.2. This is the principal robustness analysis for the direct effect. **Panel B.** E-values are computed for the controlled direct effect at each fixed value of the mediator on the risk-ratio scale and indicate the minimum strength of association, with both exposure and outcome, that an unmeasured confounder would require to account for the estimate. Lower limits are computed at the lower bound of the corresponding bootstrap confidence interval. **Panel C.** Restricted to patients in whom initial rhythm recorded by emergency medical services was classifiable; this field has been collected since 2015, so the subcohort spans 2015–2024. Initial rhythm is measured after bystander CPR has begun and may be influenced by it. It is therefore an exposure-induced confounder of the mediator–outcome relation, violating assumption A4, under which natural direct and indirect effects are not identified by the mediation formula; the estimates in this panel are reported as a conservative lower bound rather than as corrected values, and rhythm strata are not causal contrasts. A likelihood-ratio test comparing the outcome model with and without initial rhythm (main effect and its interactions) confirmed a significant improvement in fit for both outcomes (χ^2^(4) = 2391.9 for favorable neurological survival, χ^2^(4) = 2035.7 for survival to discharge; both *p* < 0.001), indicating that rhythm is strongly informative rather than a near-null addition to the model; whether the resulting shift in the estimates reflects removal of confounding, removal of genuine mediated effect through a rhythm-mediated pathway, or both cannot be distinguished from these data (Section 4). **Panel D.** Restricted to patients in the stratum without prehospital ROSC for whom the registry recorded whether spontaneous circulation returned after resuscitation in the emergency department; this field is completed only for patients who received emergency department resuscitation and was available for 47.5% of the stratum. Fixing this variable tests whether the direct component is instead transmitted through a later return of circulation. It is not, and bystander CPR did not predict it. † Computed in the whole stratum without prehospital ROSC (*n* = 70,157) rather than in the subcohort above. Survival with an unfavorable neurological outcome is survival to discharge with a Cerebral Performance Category of 3 to 5. The corresponding contrasts in the same stratum were +0.22 points (−0.14 to 0.58) for survival to discharge and +0.52 points (0.32 to 0.71) for favorable neurological survival, so the three estimates are arithmetically consistent. **Panel E.** Propensity model fitted on the same covariates as the primary analysis; stabilized weights truncated at the 1st and 99th percentiles. Before weighting, the largest standardized mean difference between exposure groups was 0.22 for calendar year, followed by witness status (0.17), age (0.12), and arrest location (0.10); after weighting, all standardized differences were below 0.02. The exposure and outcome models share the same functional form for continuous covariates, so this analysis tests whether the total effect depends on the outcome model rather than on that functional form and bears only on confounding of the exposure–outcome relation. Full balance diagnostics are given in Table S4. **Panel F.** Discharge status was unresolved for 772 patients (1.0%) who survived the emergency department and for whom no subsequent discharge, death, or second-hospital record was available; 32.0% of them had achieved prehospital ROSC, against 12.3% of the cohort, and 622 had received bystander CPR, against 150 who had not. Non-differential assignment applies the same proportion of favorable outcomes to unresolved patients in both exposure groups, and the range shown spans the full interval from none to all. Differential assignment against bystander CPR counts every unresolved patient who received bystander CPR as unfavorable and every unresolved patient who did not receive it as favorable. This is a bound rather than a plausible scenario since it requires outcome distributions in both exposure groups opposite to those observed among patients whose status was resolved. Values are point estimates obtained from the models specified for the primary analysis under each fixed assignment; bootstrap intervals were not computed for these scenarios. Bold entries are panel and section headings within the table.

**Table 5:** Effect modification of the total effect by interval from collapse to EMS activation.

Interval	*n*	Favorable Neurological Survival, Total Effect, pp (95% CI)	NNT	Survival to Discharge, Total Effect, pp (95% CI)	NNT
**Primary analysis: witnessed arrests, interval greater than zero** (*n* = 19,081)					
1 min	4328	+12.23 (9.42 to 14.95)	8	+10.45 (7.14 to 13.82)	10
2–5 min	7567	+9.17 (7.43 to 10.76)	11	+9.20 (6.96 to 11.14)	11
>5 min	7016	+3.79 (2.88 to 4.79)	26	+2.99 (1.54 to 4.21)	33
**Sensitivity: witnessed arrests, zero-minute records retained** (*n* = 33,957)					
0–1 min	19,093	+10.76 (9.41 to 12.07)	9	—	—
2–5 min	7567	+9.17 (7.20 to 10.87)	11	—	—
>5 min	7016	+3.79 (2.82 to 4.71)	26	—	—

CI, confidence interval; EMS, emergency medical services; NNT, number needed to treat; pp, percentage points. The interval was calculated as the time from the recorded collapse to the emergency call. Both timestamps derive from the national fire service record, which has been linked to the registry since 2013, so this analysis covers 2013–2024. Of 68,078 records in which both timestamps were present, 8302 (12.2%) gave a negative interval, and 4234 exceeded 120 min; both groups were excluded as internally inconsistent. A further 21,431 records carried an interval of exactly zero, in every case because the two timestamps were identical; because the primary and sensitivity analyses are themselves restricted to witnessed arrests, only the 14,876 of these that were witnessed enter the sensitivity analysis below, and it is this narrower count that appears in the “0–1 min” row. The registry manual defines the collapse time as the time at which the arrest is estimated to have occurred and directs abstractors to enter the call time when no other basis is available, so these records cannot be read as immediate notification; they are excluded from the primary analysis and retained in the sensitivity analysis, which shows the gradient is not produced by them. Within these 14,876 records, favorable neurological survival occurred in 22.1% of patients who received bystander CPR (2670 of 12,089) and 9.6% of those who did not (267 of 2787); with both exposure groups well represented across the outcome, folding them into the 0–1 min bucket does not concentrate them in a single bystander-CPR/outcome cell, consistent with the gradient being unchanged when they are retained. The analysis is restricted to witnessed arrests because, for unwitnessed arrests, the collapse time is, by construction, an estimate rather than an observed event. Models were adjusted for age, sex, arrest location, and calendar year; confidence intervals are from 400 bootstrap resamples. Likelihood-ratio tests for interaction between bystander CPR and the interval on the logit scale gave *p* = 0.87 for favorable neurological survival and *p* = 0.30 for survival to discharge. Numbers needed to treat are derived from the total effect on the risk-difference scale. The *n* shown in each panel heading is the number of patients meeting the interval criteria; the counts in each interval row are restricted to those whose discharge status could be resolved and who therefore contribute to the outcome proportions, discharge status being unresolved for 170 patients in the primary analysis, and 281 in the sensitivity analysis. Bold entries are panel headings within the table.

## Data Availability

The data underlying this study were obtained from the Korean National Out-of-Hospital Cardiac Arrest Registry, operated by the Korea Disease Control and Prevention Agency (KDCA; approved national statistics No. 117088). The data are not publicly downloadable; de-identified data are released to investigators through the agency’s standard application procedure, under which the authors obtained the data used here. The registry manual defining every variable and code is published by the agency. The classification rules applied to derive the outcome and the study population are given in full in Tables S1 and S2, and the analysis code is available from the corresponding author on reasonable request.

## Supplementary Materials

The following supporting information can be downloaded at: https://www.mdpi.com/article/10.3390/jcm15187095/s1, Table S1: Agreement between the registry’s not-applicable code and the witness-type reconstruction used to define the study population; Table S2: Classification hierarchy used to establish discharge status and neurological outcome; Table S3: Resolution of discharge status by calendar year; Table S4: Covariate balance before and after inverse probability of treatment weighting; Table S5: Events with undocumented bystander CPR status: comparison with the analytic cohort and quantitative bias analysis.
