# Supplementary material for: Do Chest Compressions Benefit the Brain Beyond Restoring Circulation? ROSC-Mediated and ROSC-Independent Pathways of Bystander CPR After Out-of-Hospital Cardiac Arrest
Source: J Clin Med. 2026 Sep 13;15(18):7095. doi: 10.3390/jcm15187095 (PMC13607137; doi:10.3390/jcm15187095)
Supplement: Supplementary file 1 [file jcm-15-07095-s001.zip › jcm-4507459-supplementary.pdf]

Supplementary Materials

Manuscript: Do Chest Compressions Benefit the Brain Beyond Restoring Circulation? ROSC-Mediated and ROSC-Independent Pathways of Bystander CPR After Out-of-Hospital Cardiac Arrest

Authors: Gyuchong Cho, Youdong Sohn

Contents

- **Table S1.** Agreement between the registry's not-applicable code and the witness-type reconstruction used to define the study population
- **Table S2.** Classification hierarchy used to establish discharge status and neurological outcome
- **Table S3.** Resolution of discharge status by calendar year
- **Table S4.** Covariate balance before and after inverse probability of treatment weighting
- **Table S5.** Events with undocumented bystander CPR status: comparison with the analytic cohort and quantitative bias analysis

Table S1. Agreement between the registry's not-applicable code and the witness-type reconstruction used to define the study population

Arrests witnessed or found by on-duty emergency medical services (EMS) personnel or health professionals were excluded from the study population, since bystander cardiopulmonary resuscitation (CPR) is not defined for such events. The registry's bystander CPR variable carries a dedicated not-applicable code for these events from 2016 onward only. For earlier years the same events were identified from the witness-type fields, which record whether the witness belonged to a listed occupation while on duty and, if so, which occupation. Both fields have been collected in every year of the registry. The exclusion was therefore defined as an on-duty witness recorded as an EMS provider or a health professional, applied uniformly across all years.

Panel A. Concordance in 2016–2024, when the registry itself assigns the not-applicable code

	Reconstruction: excluded	Reconstruction: retained	Total
**Bystander CPR coded**	32,048	0	32,048

	Reconstruction: excluded	Reconstruction: retained	Total
**not applicable**			
**Bystander CPR coded performed, not performed, or unknown**	0	251,560	251,560
**Total**	32,048	251,560	283,608

Sensitivity 100.0%; positive predictive value 100.0%. No record was classified differently by the two definitions in either direction.

Occupational categories other than EMS provider and health professional — police officers, school health teachers, transport drivers, sports facility safety officers, lifeguards, occupational safety trainees, tourism safety staff, fire safety managers, sports instructors, aviation, rail and maritime safety crew, and preschool and childcare teachers — are treated as laypersons by the registry and were retained. The registry does not assign the not-applicable code to any of these categories, and the reconstruction likewise retained them.

Panel B. Composition of the excluded events, by period

Period	Events excluded	Coded not applicable	Coded not performed	Coded performed
2008–2015	8,097	0	8,097	0
2016–2024	23,272	23,272	0	0
**Total**	**31,369**	23,272	8,097	0

Counts are of events remaining after restriction to a presumed medical cause of cardiac or respiratory origin with a recorded emergency department disposition (n = 343,913). Before 2016 the registry had no code for these events, and every one of them had been recorded as bystander CPR not performed; none had been recorded as performed.

Panel C. Proportion of events coded "not performed" that were in fact witnessed by on-duty personnel, 2009–2015

Year	Coded not performed	Of these, witnessed by on-duty EMS personnel or health professionals	%
2009	1,305	723	55.4
2010	1,786	892	49.9
2011	1,754	986	56.2
2012	2,024	1,071	52.9
2013	1,887	1,154	61.2
2014	2,180	1,241	56.9
2015	2,231	1,493	66.9
**2009–2015**	**13,167**	**7,560**	**57.4**

Panel D. Prognosis of the excluded events compared with events genuinely coded as bystander CPR not performed, 2009–2015, adults

Group	n	Witnessed arrest, %	Survival to discharge, %	Favorable neurological survival, %
Witnessed by on-duty EMS personnel or health professionals (excluded)	7,478	91.1	14.68	7.58
Bystander CPR not performed, witness not on-duty personnel (retained)	5,553	51.5	6.85	3.18

Events witnessed by on-duty personnel carried a markedly better prognosis than genuinely unexposed events. Before 2016 they were coded as bystander CPR not performed and would therefore enter the unexposed group, where they would raise its outcome rates and attenuate the estimated effect of bystander CPR. Applying the exclusion uniformly across all years removes this asymmetry and gives a population defined identically in every calendar year.

CPR, cardiopulmonary resuscitation; EMS, emergency medical services.

Table S2. Classification hierarchy used to establish discharge status and neurological outcome

The registry variable recording neurological status is defined as "neurological outcome at survival to discharge." The registry manual directs abstractors to complete it for patients whose emergency department disposition is discharge, transfer alive, or transfer during ongoing resuscitation, and for patients whose post-admission result is discharge, discharge against advice, or transfer. It is therefore recorded for patients who left the first hospital alive, whether by discharge or by transfer, and an absent value cannot be read as missing data. Discharge status was established by applying the following registry fields in a fixed order, and neurological outcome was then assigned within the resolved survivors. Both outcomes were defined across the entire cohort, with death counted as unfavorable.

Panel A. Order of application and number of patients resolved at each step

Order	Registry field	Codes applied	Assignment	n	% of cohort
1	Emergency department disposition	40 death, 41 discharge without hope of recovery	Died; unfavorable	60,784	75.24
2	Second-hospital disposition, post-admission result, or date of death	40 death, 41 discharge without hope of recovery, or any recorded date	Died; unfavorable	1,314	1.63
3	Neurological outcome at second hospital	1–5	Survived to discharge; favorable if 1 or 2, unfavorable if 3–5	1,028	1.27
4	Neurological outcome at first hospital	1–5	Survived to discharge; favorable if 1 or 2, unfavorable if 3–5	9,761	12.08
5	Outcome after admission	40 death, 41 discharge without hope of recovery	Died; unfavorable	7,124	8.82

Order	Registry field	Codes applied	Assignment	n	% of cohort
6	Date of death	any recorded date	Died; unfavorable	1	0.00
7	None of the above	—	Unresolved	772	0.96
			**Total**	**80,784**	**100.00**

Steps 2 and 3 precede the corresponding first-hospital fields because the neurological outcome field is completed for patients transferred alive as well as for those discharged, and therefore records the state at transfer rather than the final outcome. Panel C quantifies what placing them later would cost.

Panel B. Why emergency department disposition alone is insufficient

The emergency department disposition field distinguishes discharge (code 10), transfer alive (21), transfer during ongoing resuscitation (22), admission (30), death (40), and discharge without hope of recovery (41). Codes 10, 21, 22, and 30 indicate only that the patient left the emergency department alive; they do not establish survival to hospital discharge. Defining survival from this field alone would classify every admitted patient as a survivor irrespective of subsequent in-hospital death. In this cohort, step 5 identified 7,124 patients (8.8%) who left the emergency department alive but died after admission, and step 2 a further 1,314.

Panel C. Internal consistency of the neurological outcome field

Across the full registry (2008–2024, 489,689 records):

Check	Result
Records with a neurological outcome of 1–5	34,735
Of these, carrying a code for death after admission at the first hospital	0
Of these, carrying a recorded date of death at the first hospital	0
Records with an emergency department disposition of death	399,819
Of these, carrying a neurological outcome of 1–5	0
Of the records with a neurological outcome of 1–5,	18,259 (52.6%)

Check	Result
those transferred rather than discharged	
Of these, carrying an indicator of death at the second hospital	4,570

With respect to the first hospital the steps are mutually exclusive, and no record carried both a neurological outcome and an indicator of death in either direction. The field is not, however, exclusive with respect to the second hospital: because it is completed for patients transferred alive, a patient may carry a neurological outcome recorded at transfer and subsequently die at the receiving hospital.

This is why the hierarchy applies the second-hospital fields first. Had the first-hospital neurological outcome been applied before them, **753 patients carrying an indicator of death at the second hospital would have been classified as survivors** — 749 transferred alive and 4 transferred during ongoing resuscitation — and they are not evenly distributed by exposure (595 with bystander CPR, 158 without). Under that ordering, survival to discharge would rise from 13.5% to 14.5% overall and the crude difference between exposure groups from 7.9 to 8.2 percentage points. Only 19 of the 753 would have been classified as having a favorable neurological outcome, so the primary outcome is insensitive to the ordering while the secondary outcome is not.

Panel D. Patients whose status remained unresolved

All 772 unresolved patients had left the emergency department alive: 703 had been transferred alive, 26 transferred during ongoing resuscitation, 40 admitted, and 3 discharged, with no subsequent discharge, death, or second-hospital record available. Prehospital return of spontaneous circulation had been achieved in 32.0% of them, against 12.3% of the cohort, and 622 of the 772 had received bystander CPR. These patients were left unresolved rather than counted as deaths and were excluded from the principal analysis; the consequences of every

possible assignment of their outcomes are bounded in Table 4, Panel F of the main paper, and their distribution across calendar years is given in Table S3.

Table S3. Resolution of discharge status by calendar year

The fields recording outcome at a second hospital have been collected only since 2015. Patients transferred alive from the first hospital in earlier years therefore had no field from which a discharge outcome could be obtained, and their status could not be resolved. The proportion left unresolved consequently varies across the study period.

Year	Patients	Resolved using second-hospital fields	Unresolved	Unresolved, %
2009	952	0	45	4.73
2010	1,393	0	80	5.74
2011	1,545	0	82	5.31
2012	2,146	0	98	4.57
2013	2,344	0	114	4.86
2014	3,420	0	170	4.97
2015	3,533	114	50	1.42
2016	4,384	163	40	0.91
2017	5,425	206	32	0.59
2018	6,332	318	25	0.39
2019	6,457	258	24	0.37
2020	7,238	210	6	0.08
2021	8,459	251	2	0.02
2022	9,199	242	0	0.00
2023	9,267	316	3	0.03
2024	8,690	264	1	0.01
**2009–2014**	**11,800**	**0**	**589**	**4.99**
**2015–2019**	**26,131**	**1,059**	**171**	**0.65**
**2020–2024**	**42,853**	**1,283**	**12**	**0.03**
**Total**	**80,784**	**2,342**	**772**	**0.96**

From 2015 onward the second-hospital fields resolved most transferred patients, and almost none were left unresolved after 2019.

Unresolved status was slightly more frequent among patients who received bystander cardiopulmonary resuscitation (622 of 59,982, 1.04%) than among those who did not (150 of 20,802, 0.72%). The consequences of this imbalance for the estimates are examined in Table 4, Panel F of the main paper: estimates were insensitive to how many of these patients were assumed to have survived, and the direct effect on neurological outcome remained positive under every assignment examined, including one differing in direction between the exposure groups.

Table S4. Covariate balance before and after inverse probability of treatment weighting

A propensity model for bystander cardiopulmonary resuscitation was fitted by logistic regression on the same covariates used in the primary analysis. Stabilized weights were calculated and truncated at the first and ninety-ninth percentiles. Balance was assessed in the full analytic cohort (n = 80,784); outcome models were fitted in the 80,012 patients with resolved discharge status.

Panel A. Covariate balance

Covariate	Bystander CPR	No bystander CPR	Standardized mean difference
**Before weighting**			
Age, years	68.45	70.26	0.118
Male sex, proportion	0.660	0.664	0.009
Public location, proportion	0.211	0.170	0.105
Witnessed arrest, proportion	0.602	0.519	0.168
Calendar year	2019.31	2018.45	0.215
**After weighting**			
Age, years	68.92	69.13	0.014
Male sex, proportion	0.661	0.660	0.002
Public location, proportion	0.201	0.198	0.006
Witnessed arrest, proportion	0.580	0.576	0.009
Calendar year	2019.12	2019.19	0.019

Calendar year showed the largest imbalance between exposure groups before weighting, followed by witness status; all remaining covariates differed by less than 0.12. After weighting every standardized difference fell below 0.02.

Panel B. Propensity model

Term	Coefficient	Standard error	z	p
Intercept	−124.4	4.202	−29.62	< 0.001
Male sex	−0.127	0.0179	−7.08	< 0.001
Age, per year	−0.0090	0.00056	−16.00	< 0.001
Witnessed arrest	0.326	0.0163	19.97	< 0.001
Public location	0.228	0.0218	10.46	< 0.001
Calendar year	0.0624	0.00208	29.94	< 0.001

Panel C. Distribution of the stabilized weights

	Value
Range before truncation	0.498 to 2.378
First percentile	0.625
Ninety-ninth percentile	1.523

Panel D. Total effect estimated by weighting and by regression adjustment

Method	Favorable neurological survival, odds ratio (95% CI)	Survival to discharge, odds ratio (95% CI)
Regression adjustment	2.51 (2.32 to 2.72)	1.79 (1.69 to 1.89)
Weighted	2.30 (2.14 to 2.48)	1.71 (1.62 to 1.81)
Weighted with additional regression adjustment	2.48 (2.28 to 2.68)	1.79 (1.68 to 1.90)

The weighting approach relies on correct specification of the exposure model rather than of the outcome model, and therefore tests whether the total effect depends on a particular regression form. Agreement between the two approaches indicates that the total effect is not an artifact of the outcome model, but it bears only on confounding of the exposure–outcome relation by the measured covariates. It does not address the additional assumptions required to identify the mediated and direct components, and is not evidence for the direct effect.

CI, confidence interval; CPR, cardiopulmonary resuscitation.

Table S5. Events with undocumented bystander CPR status: comparison with the analytic cohort and quantitative bias analysis

Bystander CPR status was recorded as unknown for the largest single group of otherwise eligible events. The registry manual directs abstractors to enter “not performed” only where the absence of bystander CPR is explicitly documented and “unknown” where the record contains no entry, so this is not missing at random. Because the exposure is the only variable missing in these events — the mediator, the covariates, and the outcome are all recorded — the consequences of their exclusion can be bounded directly rather than only described.

Of the events remaining after restriction to a presumed medical cause of cardiac or respiratory origin, exclusion of arrests witnessed by on-duty personnel, and complete recording of the mediator, covariates, and adult age, 271,637 were eligible on every criterion other than exposure: 80,784 with bystander CPR status documented and 190,853 without.

Panel A. Characteristics and outcomes, documented versus undocumented

	Documented (analytic cohort)	Undocumented (excluded)
n	80,784	190,853
Age, mean (SD), years	68.9 (15.7)	70.7 (15.1)
Male, %	66.1	62.5
Public location, %	20.0	12.5
Witnessed arrest, %	58.0	46.4
**Prehospital ROSC, %**	**12.5**	**4.5**
**Survival to discharge, %**	**13.5**	**5.1**
**Favorable neurological survival, %**	**9.9**	**2.9**
Discharge status resolved, %	99.0	98.1

Documentation is strongly associated with prognosis: the excluded events achieved prehospital ROSC less than half as often and a favorable neurological outcome about a third as often. Selection into the analytic cohort is therefore not innocuous, and the estimates in the main paper

apply to events in which the bystander response was ascertained rather than to all EMS-assessed OHCA.

Panel B. Proportion undocumented by calendar year, region, and dispatch center

Period	Events meeting cause, disposition, and on-duty-witness criteria	Undocumented	%
2009–2012	62,062	55,593	89.6
2013–2016	73,670	59,272	80.5
2017–2020	78,963	52,628	66.7
2021–2024	85,088	48,707	57.2

Rows cover 2009–2024, the analytic period, and sum to 299,783 events of which 216,200 were undocumented; events from 2008 are not shown, prehospital ROSC not having been collected in the registry's inaugural year. Documentation improved steadily across the study period, from 97.2% undocumented in 2008 to 56.8% in 2024. Calendar year is therefore associated both with the probability of entering the cohort and, as reported in the main paper, with exposure and outcome; it is included as a covariate in every model.

Documentation also varies by locality. The tables below use a narrower denominator than the calendar-year table above: they are restricted to the 271,637 events eligible on every criterion other than exposure ascertainment — the comparison group used throughout this table — rather than to all events meeting the cause, disposition, and on-duty-witness criteria. These 271,637 events are attributed to a place of arrest recorded in one of 17 first-level administrative regions (city or province) and one of 298 fire-service dispatch jurisdictions; the tables describe how undocumented status distributes across both, descriptively rather than through a formal model, since the purpose is to establish whether non-random missingness has structure beyond calendar year rather than to adjust for it.

By region (first-level administrative division), ordered by undocumented proportion:

Region	Events eligible but for exposure ascertainment	Undocumented	%
Jeonnam (South Jeolla)	13,175	11,561	87.7
Gwangju	5,781	4,896	84.7
Gyeongbuk (North Gyeongsang)	19,175	16,191	84.4
Chungnam (South Chungcheong)	13,633	11,193	82.1
Busan	19,487	15,916	81.7
Gyeongnam (South Gyeongsang)	18,866	15,056	79.8
Ulsan	5,050	3,938	78.0
Jeonbuk (North Jeolla)	11,936	9,028	75.6
Jeju	5,886	4,440	75.4
Chungbuk (North Chungcheong)	10,816	8,131	75.2
Gangwon	11,181	8,184	73.2
Incheon	14,827	10,482	70.7
Daejeon	6,909	4,873	70.5
Gyeonggi	58,711	37,873	64.5
Sejong	814	490	60.2
Daegu	11,907	7,063	59.3
Seoul	43,483	21,538	49.5

By metropolitan status: undocumented status was somewhat more common outside the eight metropolitan cities (Seoul, Busan, Daegu, Incheon, Gwangju, Daejeon, Ulsan, Sejong) than within them — 74.5% (121,657 of 163,379) versus 63.9% (69,196 of 108,258).

By fire-service dispatch jurisdiction: among the 262 jurisdictions with at least 100 eligible events, the undocumented proportion ranged from 13.2% to 96.8% (interquartile range 64.1% to 86.1%, median 79.9%), a wider spread than across regions. This is consistent with documentation practice varying by local administrative unit rather than only by broader geography or calendar time, but jurisdiction-level variation of this magnitude could equally track systematic differences in resource levels (e.g., staffing, training, or workload in less-resourced jurisdictions) that plausibly relate to prognosis as well as to documentation; the two are not distinguishable descriptively.

This regional and jurisdictional pattern, together with the calendar-year trend above, indicates that non-random missingness in bystander CPR status has structure beyond a single national time trend. It does not, by itself, establish whether that structure correlates with unmeasured confounders of the exposure–mediator or exposure–outcome relations (assumptions A1 and A3, Section 2.6); calendar year, the covariate most directly implicated by this analysis, is already adjusted for in every model, and the bounding analysis in Panel C below does not depend on this characterization. The estimates in the main paper should nonetheless be read as conditional on an ascertained bystander-CPR status, a caveat that is discussed in the main text (Section 4) as well as here.

Panel C. Bounding the estimates over all possible exposure assignments

Each undocumented event was assigned to the exposed or unexposed group in a fixed proportion *p*, and the decomposition was repeated in the combined population of 271,637. Because the outcomes of the undocumented events are known, this is a bound on the effect of their exclusion rather than an imputation of their outcomes.

Assignment	Total effect, pp	Natural direct effect, pp	Controlled direct effect, ROSC not achieved, pp	Proportion mediated, %
**Analytic cohort (main analysis)**	**+5.24**	**+1.24**	**+0.65**	**76.4**
$p = 0$, none received bystander CPR	+4.05	+1.18	+0.79	70.8
$p = 0.10$	+3.30	+0.97	+0.60	70.5
$p = 0.20$	+2.79	+0.83	+0.49	70.3
$p = 0.30$	+2.42	+0.72	+0.40	70.1
$p = 0.50$	+1.92	+0.57	+0.28	70.1
$p = 0.742$, the proportion observed in the cohort	+1.52	+0.44	+0.17	71.3
$p = 1$, all received	+1.14	+0.21	−0.09	81.4

Assignment	Total effect, pp	Natural direct effect, pp	Controlled direct effect, ROSC not achieved, pp	Proportion mediated, %
bystander CPR				

For survival to discharge the corresponding natural direct effects were +1.44 at $p = 0$, +0.25 at $p = 0.742$, and −0.52 at $p = 1$.

The natural direct effect on the primary outcome remained positive across the entire range, from +1.18 when no undocumented event is assumed to have received bystander CPR to +0.21 when all are. The assignment implied by the registry's own coding rule is the first of these, since "not performed" is entered only when explicitly documented; consistent with that rule, the undocumented events achieved prehospital ROSC in 4.35% of cases, below the 5.88% among events explicitly recorded as not performed and far below the 14.74% among those recorded as performed. The assignment at the opposite extreme is therefore not compatible with the observed mediator distribution.

The magnitude of the total effect is, by contrast, sensitive to this assignment, ranging from +1.14 to +4.05 points against +5.24 in the analytic cohort. The proportion mediated is stable at 70% to 81% throughout.

Panel D. Inverse probability of censoring weighting

As an alternative to bounding, the analytic cohort was reweighted by the inverse probability of having bystander CPR status documented, estimated from a logistic model on the same covariates, with weights truncated at the first and ninety-ninth percentiles. This recovers the eligible population under the assumption that documentation is independent of outcome given the measured covariates — an assumption that cannot be verified and is unlikely to hold exactly.

	Total effect, pp (95% CI)	Natural direct effect, pp (95% CI)	Controlled direct effect, ROSC not achieved, pp (95% CI)	Proportion mediated, %
Favorable neurological survival	+4.16 (3.87 to 4.52)	**+0.95 (0.72 to 1.19)**	**+0.53 (0.33 to 0.73)**	77.1 (72.7 to 81.7)
Survival to discharge	+4.47	+0.72	+0.37	—

Confidence intervals for the primary outcome are from 300 bootstrap resamples in which the censoring model was refitted within each resample.

Estimated probabilities of documentation ranged from 0.058 to 0.767, so the weights are unstable at the extremes and this analysis should be read alongside the bounds in Panel C rather than in place of them. Under weighting the direct component is attenuated relative to the analytic cohort but retains its sign and magnitude.

CPR, cardiopulmonary resuscitation; EMS, emergency medical services; pp, percentage points; ROSC, return of spontaneous circulation; SD, standard deviation.
